# Empathy, Guilt Proneness, and Gender: Relative Contributions to Prosocial Behaviour

**DOI:** 10.5964/ejop.v12i2.1097

**Published:** 2016-05-31

**Authors:** Linda Torstveit, Stefan Sütterlin, Ricardo Gregorio Lugo

**Affiliations:** a Department of Psychology, University of Copenhagen, Copenhagen, Denmark; b Section of Psychology, Lillehammer University College, Lillehammer, Norway; University College Cork, Cork, Ireland

**Keywords:** guilt proneness, guilt, empathy, personality, prosocial behaviour, helping behaviour, gender

## Abstract

Guilt is a moral emotion that is often looked upon as a negative trait. However, studies show that some individuals are more predisposed to think, feel and act in a more ethical manner because of a lower threshold to experience guilt. Some theories of helping behaviour emphasize the evolutionary mechanisms, while other theories stress the importance of social variables. This study investigated whether guilt proneness as a dispositional trait can be associated with prosocial behaviour. Five hundred sixty-nine participants reported in an online survey their own levels of guilt proneness, frequency of prosocial behaviour, and related cognitions such as empathy. This study is among the first to demonstrate how guilt proneness combined with empathy can explain additional variance in prosocial behaviour. The findings also indicate gender differences in the precursors of prosocial behaviour, suggesting women are more influenced by the effects of guilt proneness on prosocial behaviour than men.

During the past two decades there has been an extensive amount of research examining the role guilt plays in our cognitions and actions. Guilt can have an impact on human actions and may sometimes motivate good deeds (Estrada-Hollenbeck & Heatherton, 1998). Research indicates a positive correlation between personal experience of or perceived guilt and helping behaviour (Miller, 2010). The feeling of guilt may occur when someone feels responsible for another person’s negative state (Estrada-Hollenbeck & Heatherton, 1998). Prosocial or helping behaviour is voluntary behaviour intended to benefit another person. This is behaviour characterized by concern about rights, feelings and well-being of others (Estrada-Hollenbeck & Heatherton, 1998). Altruism, a related concept, is the practise of selfless concern for or devotion to the welfare of others. When a motivation for prosocial behaviour is to help others without any thought of what one might get in return, we generally talk about altruism (Hogg & Vaughan, 2011).

Recent research suggests there may be certain individuals that are predisposed to act more ethically than others even when wrongdoing is private (Cohen, Panter, & Turan, 2012). These studies propose that some individuals share an inter-individual trait called guilt proneness (Cohen et al., 2012). Guilt proneness is a personality characteristic related to “a predisposition to experience negative feelings about personal wrongdoing, even when the wrongdoing is private” (Cohen et al., 2012, p. 2). There may be many reasons behind a prosocial act. The feeling of guilt before a transgression has taken place may be explained by the skill of the individual to anticipate consequences or to think before acting to a greater degree than others (Cohen et al., 2012). However, guilt proneness may predispose the individual to think, feel and act ethically and therefore might have an effect on helping behaviour. The trait is often characterized by the expectation of a negative feeling before committing a transgression, rather than by guilty feelings in a particular moment as various studies have examined (Miller, 2010; Roberts, Strayer, & Denham, 2014).

Although guilt and guilt proneness are closely connected there is a difference between them that needs to be emphasized. Guilt is an uncomfortable moral emotion that occurs when a person has transgressed a social norm. Guilt proneness on the other hand is a personality trait that involves a lower threshold of experiencing guilt even when a transgression is private, or before the individual has done a transgression (Cohen et al., 2011; Miller, 2010). Previous studies on guilt proneness indicate that there is a significant difference between men and women in levels of this personality trait. Some studies suggest that the difference in socialization of boys and girls may impact the threshold of experience guilt (Benetti-McQuoid & Bursik, 2005; Cohen et al., 2011; Gilligan, 1977; Katchadourian, 2010; Tangney, 1991). Further research is needed to replicate these findings in different samples.

The opportunity to help or encourage others arises daily, such as by helping an elderly person across a street, giving a pregnant woman a seat on the bus or donating blood. Much of the earlier research has looked at the phenomena at a group-level (Darley & Batson, 1973; Darley & Latané, 1968; Markey, 2000). However, as other studies have shown helping behaviour at an individual level can be just as important since it can play a mediating role in environmental influences on behaviour (Cohen et al., 2012; Tangney & Dearing, 2002; Tangney, Stuewig, Mashek, & Hastings, 2011; Zemack-Rugar, Bettman, & Fitzsimons, 2007).

The gap between perceiving a situation where someone needs help and taking action may be affected by multiple factors, such as diffusion of responsibility, perceived personal risk or conflict with other goals (Darley & Batson, 1973). In this context it seems reasonable to investigate associated cognitions such as empathy). Evidence from a study on emotions and prosocial behaviour suggest that empathic children score higher on adaptive feeling of guilt (Roberts et al., 2014). Empathy is the experience where one understands and feels another person’s emotions and it is closely linked with both guilt proneness and prosocial behaviour (Estrada-Hollenbeck & Heatherton, 1998; Larsen, Buss, & Wismeijer, 2013).

## Current Study

The focus of this study was to examine the relationship between helping behaviour and guilt proneness. The hypothesis builds on the assumption that more guilt prone individuals may be predisposed to think, feel and act in a more ethical way, thus exert more helping behaviour than people with low guilt proneness. Since empathy is closely linked to prosocial behaviour, another hypothesis tested was that guilt proneness adds additional power to the prediction of prosocial behaviour and thus improves classical models relying on empathy alone. While several previous studies showed that females score on average higher on guilt proneness (Cohen et al., 2011; Tangney, 1991; Wong, 2003), the implications of these findings for associations with resulting prosocial behaviour remain unclear. We therefore hypothesized that gender differences in guilt proneness might contribute to the explanation of prosocial behaviour.

## Methods

### Research Design and Ethics

Data used in this paper is based on self-report data collected by means of an online survey conducted with the software package *SoSciSurvey* (Leiner, 2014). The survey has been approved by the Norwegian Social Science Data Protection Authority (NSD; project nr. 40376; http://pvo.nsd.no/prosjekt/40376).

### Participants

To increase generalizability, the sample of 568 participants was recruited internationally through social media, psychological forums such as Psychological Research on the Net (Krantz, 2015), from an Upper Secondary School in Norway and different International Colleges and Universities (United Kingdom: University of Greenwich, USA: Carleton College, St. Louis/MN, Macalester College, St. Paul/MN; Presbyterian College, Clinton/SC; Poland: University of Gdansk; Germany: University of Jena, University of Würzburg; Netherlands: University of Twente). The questionnaire had two language options Norwegian and English. The Norwegian language option was chosen by 345 (60.7%) respondents, while 223 (39.3%) participants responded in English. Of them 396 (69%) were women. The average age was 30 (*M* = 30.32, *SD* = 13.33), with an age-range of 15-76.

### Material

#### Guilt and Shame Proneness Scale

Guilt proneness was measured by using the Guilt and Shame Proneness Scale (GASP; Cohen, Wolf, Panter, & Insko, 2011). This scale was based on another test called Test of Self Conscious Affect (TOSCA; Tangney & Dearing, 2002). GASP is a scale consisting of four subscales: Guilt-Negative-Behaviour-Evaluation, Guilt-Repair, Shame-Negative-Self Evaluation, and Shame-Withdraw. The subscale Guilt-Negative-Behaviour-Evaluation was the only subscale used as a measure of guilt proneness, since the focus in this project was to see how respondents evaluate themselves and their behaviour, not on how they compensated for the transgression (guilt-repair). Respondents imagine themselves in situations where they have committed transgression and are then asked to indicate the likelihood they would act or feel in the way described. The scale goes from (1) *very unlikely* to (7) *very likely.* An example of an item used in this subscale is ”You lie to people, but they never find out about it. What is the likelihood that you would feel terrible about the lies you told?” The benchmark on the test quality parameter is set on .60 of each subscale and the subscale has shown to be reliable (Cronbach’s α = .69; Cohen et al., 2011).

#### Altruistic Personality Scale

Altruistic Personality Scale was used to measure the self-reported tendency and frequency participants engage in altruistic acts or helping behaviour (APS; Rushton, Chrisjohn, & Fekken, 1981). This is a 20-item, 5-point scale, ranging from (1) *never* to (5) *very often.* The following are examples of items: “I have given directions to a stranger. I have done volunteer work for a charity. I have donated blood. I have offered my seat on a bus or train to a stranger who was standing”. The scale is highly reliable (Cronbach’s α = 0.9; Rushton et al., 1981).

#### Brief Social Desirability Scale

When studying topics of helping behaviour one might encounter the problem of social desirability. Social desirability refers to the respondent’s tendency to give desirable responses instead of indicating what they really do, feel or think (Hayes, 2000). A measure to address this particular issue is Brief Social Desirability Scale (BSDS; Haghighat, 2007). An item in this scale may be: “would you ever lie to people?”. The scale has satisfactory reliability (α = .60; Haghighat, 2007) and was used to segregate responses of highly desirable responses from the reliable responses.

#### Toronto Empathy Questionnaire

To assess the level of empathy Toronto Empathy Questionnaire was used (TEQ; Spreng, McKinnon, Mar, & Levine, 2009). This test consists of 16 items, each is rated on a 5-point scale from (0) *never* to (4) *always*. “I get a strong urge to help when I see someone who is upset” is an example of an item. Cronbach’s α for the scale is .85 (Spreng et al., 2009).

### Statistical Analysis

All statistical analysis was done by SPSS (Version 22.0). Statistical requirements for multivariate testing were checked before statistical analysis. Assumption of normality was checked via Shapiro-Wilks test. Homogeneity of variance was tested via Levene’s test. A bivariate Pearson’s correlation was calculated for all relevant variables. A stepwise regression analysis will show the influence of empathy and guilt proneness on prosocial behaviours. Gender specific effects will also be investigated.

## Results

### Reliability Analysis

Normality and homogeneity of variance was ensured. In this study, all scales except the Brief Social Desirability Scale (BSDS, α = .38) can be considered reliable. BSDS was not included in further analysis. The subscale of Guilt Proneness showed a test quality parameter of .60. The measure of prosocial behaviour, APS, had a Cronbach’s Alpha of .86, while the Toronto Empathy Questionnaire had a reliability of .78.

### Gender, Empathy, Guilt Proneness and Prosocial Behaviour

Consistent with earlier research examining the personality trait guilt proneness (Cohen et al., 2012) there was a correlation between guilt proneness and empathy. Correlations indicated lower levels of guilt proneness and empathy *r*(373) = -.285, *p* < .01 in males. There was also a positive correlation between guilt proneness and prosocial behaviour, which supports one of the research hypotheses. Prosocial behaviour did, in this study, significantly correlate with all investigated variables, but was not associated with gender (see Table 1.)

**Table 1:** Correlation Between Variables

Variables	1	2	3	4
1. Gender	-	-.309*	.000	-.285*
2. Guilt Proneness		-	.207*	.273*
3. Prosocial Behaviour			-	.239*
4. Empathy				-

*Note*. In Gender, 0 = female, 1 = male.

*p < .01.

A stepwise regression analysis was used to test a model to predict frequency of self-reported prosocial behaviour from the scores of empathy and guilt proneness. The two variables were entered in the regression analysis, whereas empathy was entered in Step 1, explaining 6% of the variance of prosocial behaviour. After the entry of empathy and guilt proneness in Step 2 the total variance explained by the model as a whole was 8%, *F*(1, 356) = 15.24, *p* < .001, *R^2^*_change_ = .02, *F*_change_(1, 356) = 15.24, *p* < .001. The empathy score demonstrates a standardized beta value of β = .98 (*p* < .01), while the score of guilt proneness showed a standardized beta coefficient of .153 (*p* < .01).

When separated by gender the model was significant for females showing a significant prediction of prosocial behaviour by empathy alone (Model 1) and a significant improvement of explanatory power after addition of guilt proneness as additional variable (Model 2) as you may see in Table 2. For male participants, the additional predictor guilt proneness did not improve the regression model of empathy on prosocial behaviour as Table 3 represent. The overall statistical model remained significant after entering guilt proneness.

**Table 2:** Stepwise Regression on Prosocial Behaviour in the Female Sample. Guilt Proneness Predicts Additional Variance Beyond Empathy.

Variable	*B*	β	*t*	*p*
Model 1				
Empathy	.439	.223	3.70	<.001
Model 2				
Empathy	.387	.197	3.32	.001
Guilt Proneness	.363	.139	2.28	.024

*Note.* Model 1: *R*^2^_change_ = .050; *R*^2^_adj_ = .050. Model 2: *R*^2^_change_ = .019; *R*^2^_adj_ = .061. Total *R*^2^_adj_ = .061.

**Table 3:** Stepwise Regression on Prosocial Behaviour in the Male Sample. Guilt Proneness Does not Predict Additional Variance Beyond Empathy.

Variable	*B*	β	*t*	*p*
Model 1				
Empathy	.730	.367	3.81	<.001
Model 2				
Empathy	.618	.311	3.12	.002
Guilt Proneness	.405	.188	1.88	.063

*Note*. Model 1: *R*^2^_change_ = .135; *R*^2^_adj_ = .126. Model 2: *R*^2^_change_ = .032; *R*^2^_adj_ = .149. Total *R*^2^_adj_ = .149.

In females, empathy explained 4.6% of frequency of prosocial behaviour, when guilt proneness is added the model improves. Empathy combined with prosocial behaviour could explain 6% of the variance of prosocial behaviour in the female sample. However, in the male sample guilt proneness did not have any additional effect over empathy on helping behaviour as it had in the female part of the sample. However, the results indicate that the model is better fitted for males (*R*^2^_adj_ = .149) than for females (*R*^2^_adj_ = .061).

There was a wider distribution in reports of helping behaviour in men (*SD* = 13.2) compared to women (*SD* = 11.7), however this difference was not significant (*t*(483) = .010, *p* = .992). This is in line with previous research applying the Altruistic Personality Scale (Rushton et al., 1981).

There are also some findings of ceiling effects in the guilt proneness responses. Especially women tend to report greater levels of guilt proneness. The distribution of women`s responses tends to gather in the high end of the guilt proneness scale, whereas there is a more variance and distribution in the men's responses.

## Discussion

Most people have the capacity to experience guilt, an uncomfortable feeling of having committed a transgression. It is regarded as a negative emotion, and it is often associated with mental disorders, such as major depression, and preservative thinking (Nolen-Hoeksema, 2014). However, the experience of guilt has an adaptive function that may help individuals to regulate themselves in order to decrease the possibility of future guilt experiences (Izard, 1977; Tomkins, 1963). Moral emotions are adaptive for functioning and social cohesion, motivating individuals to make up for incorrect behaviours or to keep up with one’s standards. Some individuals have a greater capacity to experience guilt even when a transgression is private and these people are considered to have higher levels of guilt proneness (Cohen et al., 2011).

### Guilt Proneness and Prosocial Behaviour

In this study guilt proneness was associated with prosocial behaviour or helping behaviour, which is consistent with previous research (Benetti-McQuoid & Bursik, 2005; Cohen et al., 2011). Zemack-Rugar et al. (2007) reported similar findings showing helping behaviour was more likely in high guilt prone individuals compared to participants low in guilt proneness. In their study subjects were asked to do a tedious task to assist a charity. The time the participants were willing to spend on it was measured. Findings suggested that highly guilt prone individuals spent more time on volunteering than individuals low in guilt proneness (Zemack-Rugar et al., 2007).

Different studies suggested that guilt proneness is related to different moral behaviours such as law abiding, altruistic behaviour, conscientiousness, agreeableness, empathy, perspective taking and self-actualizing (Cohen et al., 2011; Tangney & Dearing, 2002; Tangney et al., 2009; Wong, 2003). Overall, these qualities may increase the likelihood of more helpful behaviour and guilt proneness may also explain why an individual feel more responsibility for another person’s well-being (Miller, 2010). In the current study there are indications that prosocial behaviour can be explained by empathy, a finding that is consistent with previous studies (Cohen et al., 2011, 2012; Tangney & Dearing, 2002).

Guilt proneness is characterized by a great sense of responsibility to others (Schaumberg & Flynn, 2012). When a guilt prone individual perceives someone in need of help, they may feel a greater responsibility to act. Once a guilt prone individual perceives or thinks they have failed or transgressed a norm or their own standards, they might feel a sense of increased obligation (Schaumberg & Flynn, 2012). Ferguson, Stegge, and Damhuis (1991) suggest that the emergence of guilt proneness depends on a variety of socialization experiences combined with the child’s cognitively based skills such as empathy or sense of responsibility. Guilt prone individuals do not need anyone else to prevent them for committing moral transgressions. Their conscience activates moral cognitions and this may prevent them to continue with behaviours that are incongruent with their standards (Cohen et al., 2012).

Research on guilt proneness is relevant not only in the context of prosocial behaviour, but also in the field of criminology and to the rehabilitation process. Tangney et al. (2011) conducted a study of 550 jail inmates on guilt proneness. Results showed a substantial variation in guilt proneness among the inmates that was related to risk factors such as criminal history, psychopathy, violence and antisocial personality disorder. Guilt proneness appears to be a protective factor and was related with lower levels of antisocial personality and psychopathy. Inmates high in guilt proneness tend to show a smaller severity of criminal charges and fewer prior felony convictions (Tangney et al., 2011). A longitudinal study showed that individuals high in guilt proneness were less likely to try drugs and alcohol, less likely to become a criminal and less likely to commit suicide (Tangney & Dearing, 2002). Consistent with these findings other researchers also propose that guilt proneness can promote prosocial behaviours and inhibit aggressive behaviours, which may support the findings in this study (Ferguson et al., 1991).

This research has shown that empathy affects prosocial behaviour separately from guilt proneness even if they are directly correlated. Prosocial behaviours may be dependent on empathic cognitions as well as guilt proneness.

### Gender Differences in Prediction of Prosocial Behaviour

The results from our study are consistent with previous research that has associated capacity to experience guilt with empathy (Cohen et al., 2011; Tangney, 1991). In the socialization of girls, the focus is on how to comply with friends, to make amends after a fight and to take responsibility of others well-being (Benetti-McQuoid & Bursik, 2005). Parental styles are influenced by the child’s gender. Research have proposed that boys develop a higher threshold of sensitivity to other’s emotions and therefore may be to a lesser degree consciously aware of the impact on their behaviour on others’ well-being (Gilligan, 1977; Robertson, Snarey, & Ousley, 2007). During upbringing, children learn about gender roles and what is expected of them. Boys are nurtured to be more assertive, while girls are encouraged to be more caring (Benetti-McQuoid & Bursik, 2005; Hogg & Vaughan, 2011; Kochanska, Gross, Lin, & Nichols, 2002). These findings provide a plausible framework for the explanation of gender differences in the association of guilt proneness and prosocial behaviour.

According to Gilligan (1982), women are brought up to care for and be morally responsible for others, while men are raised to follow an ethic of righteousness and justice. This upbringing for men creates autonomy and some sort of separation of one’s moral judgment of others. On other hand ethics of care emerges from a sense of responsibility. Gilligan (1982) suggests that boys are more likely to experience guilt for violating rules, while girls experience more guilt if their transgression is damaging their relation to others.

Another possible explanation of differences in the relation between guilt proneness, empathy and helping behaviour between genders could be that because of socialization, women tend to have an enhanced capacity to be aware and be attuned to the effects of their behaviour, that they might anticipate other people’s reaction to their actions. This is closely linked with guilt proneness and empathy (Benetti-McQuoid & Bursik, 2005).

Katchadourian (2010) argues that another important factor that leads women to be more guilt prone can be that they are brought up to be more sensitive to others feelings and well-being. Men and women are as equally likely to observe a situation where someone needs help or comfort. However, women are more likely to be distressed and to express concern for that person than men. This can in turn be a possible explanation of why females have a lower threshold for feeling guilty (Katchadourian, 2010).

A lower threshold to guilt has been associated with empathic responsiveness and perspective taking, whereas female students were more altruistic to first kin or close relatives (Wong, 2003). This finding shows the complexity of the relation between guilt, empathy, helping behaviour, culture and biological explanations such as kin selection.

Findings from our study show similar results as previous research. The model shows better predictive effect sizes in males, but does not reach statistical significance (*p* = .063). Guilt proneness combined with empathy can explain 14% of the variance in helping behaviour in men. In females these variables can only explain 6%. These findings suggest that there are more variables needed to explain helping behaviour in females. Future research on gender differences should therefore account for potentially different degrees of complexity when it comes to the prediction of prosocial behaviour.

Prosocial behaviour is a complex behaviour and can be explained by multiple factors such as empathy, attention, attribution, perceiving a situation where someone needs help, have the opportunity to help, consideration of the risks of getting involved and social norm theory, to mention a few. Even though the findings indicate a gender difference that is consistent with earlier findings, it is necessary to emphasize the importance of individual differences, meaning that the gender differences should not be generalized. Cognitions, emotions and behaviour of men and women overlap in many ways (Katchadourian, 2010).

### Limitations and Implications for Future Research

Although the findings support the main hypothesis of a relation between guilt proneness and prosocial behaviour, the completion rate is 73% and thus self-selection biases cannot be excluded. Among the disadvantages in online surveys one may find transparency in process of responding, poor response rates and higher probability for dropouts. The strengths of online surveys can be that it is more convenient for the participants, there is more heterogeneous samples, have a lower probability of social desirability due to their anonymity and allows for international recruitment and thus more heterogeneous and generalizable samples.

Another methodological weakness in this study is concerning the convenience sample used in this project. Sociodemographic information on geographical location, nationality/ ethnicity and native language is missing. Studies show that culture may have an effect on guilt proneness (Wong, 2003), therefore it would be appropriate to have an overview of different countries or cultures the participants are from. However, the study shows normal distributions in all variables regardless of cultural background.

The survey consisted of a social desirability scale (BFSD; 2007), which was intended to identify responses affected by eagerness to please the researchers or to be perceived as more prosocial than one may be in reality. When dealing with a highly socially desirable behaviour such as prosocial behaviour one will most likely meet social desirability in the participants’ reports. However, there is more anonymity present in an online survey, which means that one can assume a lesser degree of social desirability.

Another limitation in this study was that Guilt Proneness Scale did show ceiling effects in females. A plausible explanation for this could be that in females there are perhaps needed more items to observe more variation, than only four items. Future research should try to avoid this, perhaps by adding another comprehensive measure of guilt proneness to ensure variation. Ceiling effects can skew the results leading to possible type I errors.

As in all correlational studies, the question of causality remains. Future research should aim to corroborate the presented findings in an experimental setting, since self-reports could be affected by social desirability. An experimental setting would also be able to investigate how the relation found in this study is affected by situational factors.

## Conclusion

Normally, experience of guilt is regarded as something negative, however previous research has shown indications of the importance of guilt proneness in moral behaviour. Some research has studied how guilt proneness relates to unethical behaviour (Cohen et al., 2012; Tangney et al., 2011). Since many established theories of prosocial behaviour, such as social norm theory and social exchange theory, are group - based theories, this project aimed to investigate how a dispositional factor within an individual could relate to self-reported helping behaviour.

The original research hypothesis was confirmed and the findings demonstrate that there is an association between guilt proneness and prosocial behaviour. This study is among the first to demonstrate how guilt proneness combined with empathy explained additional variance in prosocial behaviour. The findings suggest that there are gender differences in guilt proneness explaining prosocial behaviour. Although women tend to be more guilt prone as the findings suggest, the model works better on males, whereas 14% of the variance could be explained by the two variables (empathy and guilt proneness) in males. To explain prosocial behaviour in women, more determinants of behaviour might be needed, and this might be a possible aim for future research.
